# Functional Autoantibodies Targeting G-Protein-Coupled Receptors and Their Clinical Phenotype in Patients with Long-COVID

**DOI:** 10.3390/ijms26146746

**Published:** 2025-07-14

**Authors:** Sophia Hofmann, Marianna Lucio, Gerd Wallukat, Jakob Hoffmanns, Thora Schröder, Franziska Raith, Charlotte Szewczykowski, Adam Skornia, Juergen Rech, Julia Schottenhamml, Thomas Harrer, Marion Ganslmayer, Christian Mardin, Merle Flecks, Petra Lakatos, Bettina Hohberger

**Affiliations:** 1 Department of Ophthalmology, Universitätsklinikum Erlangen, Friedrich-Alexander-Universität Erlangen-Nürnberg, 91054 Erlangen, Germany; 2 Research Unit Analytical BioGeoChemistry, Helmholtz Zentrum München-German Research Center for Environmental Health, 85764 Neuherberg, Germany; 3 Berlin Cures GmbH, 13125 Berlin, Germany; 4 Rheumatology and Immunology Section, Department of Internal Medicine 3, Universitätsklinikum Erlangen, Friedrich-Alexander-Universität Erlangen-Nürnberg, 91054 Erlangen, Germany; 5 Deutsches Zentrum für Immuntherapie (DZI), Universität of Erlangen-Nürnberg, Friedrich-Alexander-Universität Erlangen-Nürnberg, 91054 Erlangen, Germany; 6 Infectious Disease and Immunodeficiency Section, Department of Internal Medicine 3, Universitätsklinikum Erlangen, Friedrich-Alexander-Universität Erlangen-Nürnberg, 91054 Erlangen, Germany; 7 Department of Internal Medicine 1, Universität of Erlangen-Nürnberg, Friedrich-Alexander-Universität Erlangen-Nürnberg, 91054 Erlangen, Germany

**Keywords:** functional autoantibodies, Long-COVID, symptoms, Post-COVID syndrome, phenotype, autoantibodies, G-protein-coupled receptors, beta-adrenergic receptor

## Abstract

Long-COVID (LC) is characterized by diverse and persistent symptoms, potentially mirroring different molecular pathways. Recent data might offer that one of them is mediated by functional autoantibodies (fAAb) targeting G protein-coupled receptors (GPCR). Thus, the aim of this study was to investigate the clinical phenotype of patients with LC in relation to their GPCR-fAAb seropositivity. The present study recruited 194 patients with LC and profiled them based on self-reported symptoms. GPCR-fAAb seropositivity was identified by using a cardiomyocyte bioassay, testing the presence and functionality of the AAbs. Logistic regression, clustering, and decision tree analyses were applied to examine associations between GPCR-fAAb profiles and self-reported symptoms considering age and gender. The most prevalent GPCR-fAAbs in patients with LC were fAAB targeting the β2 adrenergic receptor (β2-fAAb, 92.8%), the muscarinergic M2 receptor (M2-fAAb, 87.1%), the Angiotensin II type 1 receptor (AT1-fAAb, 85.6%), and angiotensin (1–7) Mas receptor (MAS-fAAb, 85.6%). β2-fAAb showed a significant relation with dizziness, lack of concentration, and POTS, while Endothelin Type A receptor functional autoantibody (ET-A-fAAb) was significantly related to deterioration of pre-existing neurological disorders. Statistical analysis indicated a strong positive correlation between M2- and β2-fAAb; as in addition, an association of β2-fAAb and gender was observed to one of the major clinical symptoms (fatigue/PEM), a critical impact of GPCR-fAAb on LC-pathogenesis can be assumed. Summing up, the present data show that specific GPCR-fAAb are associated with distinct clinical phenotypes. Especially, the combination of M2- and β2-fAAb seemed to be essential for the LC-phenotype with a combination of fatigue/PEM and lack of concentration as major clinical symptoms.

## 1. Introduction

Long/Post-COVID Syndrome (LC/PCS) is characterized by persisting or novel clinical symptoms after an acute infection with the severe acute respiratory syndrome coronavirus 2 (SARS-CoV2) virus, causing Coronavirus Disease 2019 (COVID-19) [1]. LC prevalence varies widely, being dependent on i.a. risk factors and vaccination status. [2,3]. As many as 65 million people were estimated to be affected with LC worldwide [4]. Consequently, LC has the potential to impact the quality of life of each patient, health care systems and economy [5].

The diverse and varying LC symptoms argue for different molecular pathways, which mediated LC pathogenesis. Several patients suffer at clinical symptoms, caused by specific restrictions and dysfunctions of distinct organ systems (e.g., heart, lung) [5,6,7,8] being objectively visible on, e.g., X-ray, while some patients complain about dominantly psychological impairments after COVID-19 [9]. Additionally, patients report of post-exertional malaise (PEM), neurological impairments, including cognitive symptoms (e.g., impairment of memory issues, attention deficits, brain fog), disrupted sleep patterns, loss of hair, taste or smell, postural orthostatic tachycardia syndrome (POTS) and dysautonomia [10,11,12,13,14,15,16,17,18,19,20]. The precise underlying molecular mechanisms of the diverse clinical phenotypes in the context of LC are elusive. Researchers propose that next to viral persistence, a hyperstimulation of the immune system or molecular mimicry might induce the generation of autoantibodies (AAb) targeting a variety of receptors, involved in the regulation of the autonomic nervous system, immune modulation and vascular response [21,22,23,24,25]. Recent data suggest an involvement of AAb targeting G protein-coupled receptors (GPCR-AAb) in LC pathogenesis. Interestingly, a subgroup of GPCR-AAb with functional activity (GPCR-fAAb) were observed in sera of patients with PCS [26]. In contrast to well-known AAb, which primarily trigger immune responses, destroying their targets and/or associated tissues, GPCR-fAAb do not consistently lead to apoptosis or inflammation in cells, tissues or organs. Instead, these ‘functional autoantibodies’ initiate uncontrolled signaling cascades via hyperactivation of their target receptors with consecutive prolonged desensibilization of them [27]. This activation is hypothesized to lack regulatory mechanisms, such as receptor downregulation, thus inducing a pathological cellular cascade [28].

Previously these GPCR-fAAb were observed in sera of patients with different disorders, sharing a vascular component and no causal therapeutic option. Research data showed that *Trypanosoma cruzi*, the pathogen responsible for Chagas’ Cardiomyopathy, triggers a functional autoimmune response against the human β1adrenergic receptor (β1-fAAb) in the cardiovascular system, mediated by molecular mimicry [29]. Additionally, a seropositivity of β2-fAAb was also observed in sera of patients with myocarditis, hypertension, POTS, glaucoma, allergic asthma and chronic fatigue syndrome (CFS) [27,30,31,32,33,34,35,36]. Of interest, the coexistence of β2-fAAb and muscarinergic M2 receptor (M2)-fAAb has also been observed in sera of patients with POTS, dysautonomia and PCS, respectively [26,37]. It can be hypothesized that different GPCR-fAAb pattern might trigger different clinical phenotypes. Thus, it was the aim of the present study to investigate the phenotype of patients with LC and a seropositivity of GPCR-fAAb considering the different GCPR-fAAb patterns in comparison to controls.

## 2. Results

**Figure 1:**
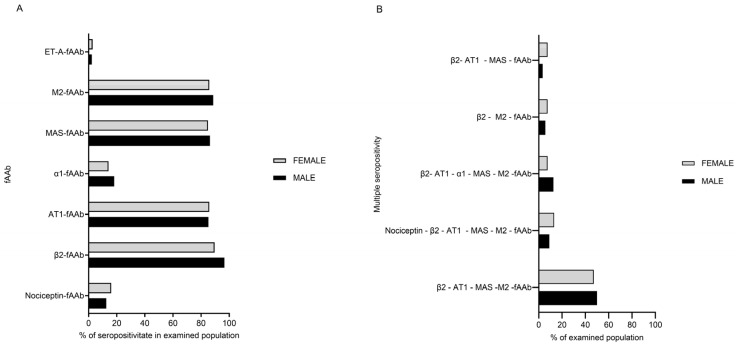
Seropositivity of GPCR-fAAb (**A**) and distinct patterns of combined seropositivity (**B**) of patients with LC. The majority demonstrated seropositivity for the following antibodies: β2-fAAb (92.8%), M2-fAAb (87.1%), AT1-fAAb (85.6%), and MAS-fAAb (85.6%). By contrast, α1-fAAb was present in 16.0%, while Nociceptin-fAAb and ET-A-fAAb were observed in 14.4% and 2.6%, respectively. The most frequent combination was M2-, MAS-, AT1- and β2-fAAb (47.1% in females and 50% in males). The combination of a seropositivity of Nociceptin-, AT1-, MAS- and M2-fAAb was observed in 13.2% of females and 9% of males LC patients. Seropositivity for a combination of β2-, M2-, AT1- and MAS-fAAb was observed in 7.5% of females and 12.5% of males; β2-M2-fAAb double seropositivity was observed in 7.5% of females and 5.6% of males. The least common combination was β2-, AT1- and MAS-fAAb (7.5% of females, 3.4% of males).

GPCR-fAAb seropositive patients with LC showed different patterns of GPCR-fAAb (Figure 1). Most patients with LC showed a seropositivity of β2-fAAb (92.8%), M2-fAAb (87.1%), AT1-fAAb (85.6%), and MAS-fAAb (85.6%). Seropositivities of α1-fAAb (16.0%), Nociceptin-fAAb (14.4%), and ET-A-fAAb (2.6%) were a less common feature in the study cohort. There was no gender effect across the different GPCR-fAAbs (Figure 1A).

**Figure 2:**
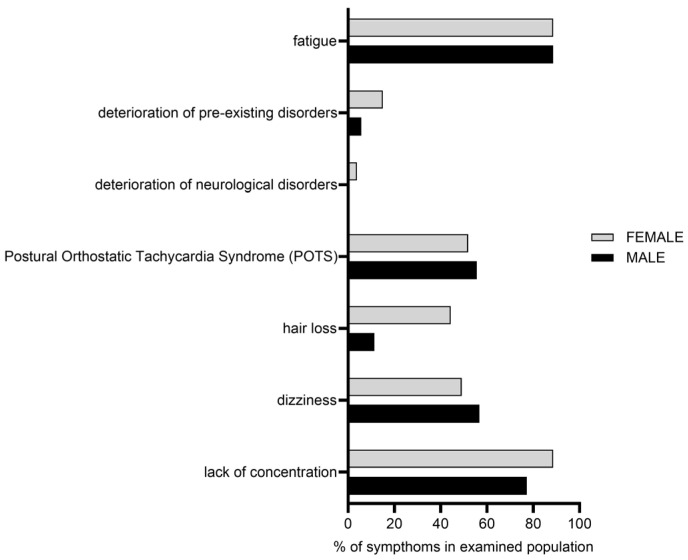
Self-reported clinical symptoms of the patients with LC. The most commonly self-reported symptoms were ‘fatigue/post-exertional malaise (PEM)’ (female: 88.6% and male: 88.6%) and ‘lack of concentration’ (female: 88.6% and male: 77.2%). Additional self-reported symptoms were ‘dizziness’ (female: 49% and male: 56.8%), ‘Postural Orthostatic Tachycardia Syndrome’ (POTS) (female: 51.8% and male: 55.6%), and ‘hair loss’ (female: 44.3% and male: 11.3%). A subset of participants (female: 15.1% and male: 5.5%) reported a worsening of pre-existing medical conditions, with neurological disorders being the most frequently mentioned (female: 3.7% and male: 0%).

The frequency of the self-reported symptoms in this study cohort is visualized in Figure 2. ‘Fatigue/PEM’ (88.7%), ‘lack of concentration (88.7%)’ and ‘POTS (53.6%)’ were the most common ones, reported by the patients with LC. The frequencies of the other self-reported symptoms were ‘dizziness’ (47.4%), ‘hair loss’ (29.4%) and ‘deterioration of pre-existing disorders’ (10.8%, especially neurological disorders, 2.1%). A gender effect was observed for clinical symptom ‘lack of concentration’ (*p* = 0.0133, CI: [1.25, 6.89]; OR = 2.94), ‘hair loss’ (*p* = 2.23 × 10^−6^, CI: [3.14, 15.88]; OR = 7.06) and ‘deterioration of pre-existing disorders’ (*p*-value = 0.049854; CI: [1.00, 9.88]; OR = 3.14).

**Figure 3:**
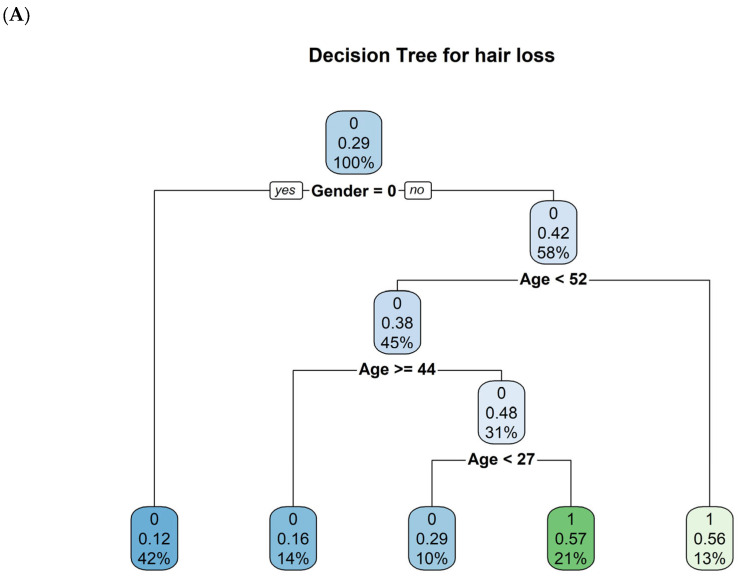

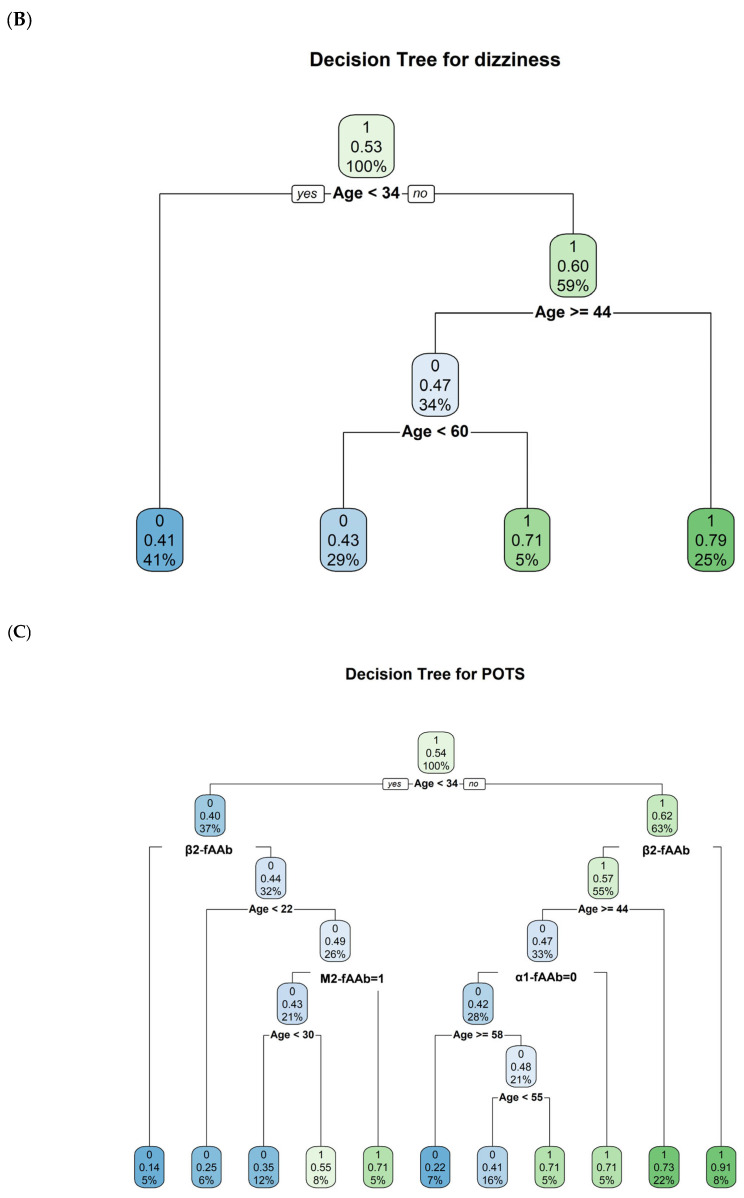
Decision tree for the self-reported clinical symptoms: (**A**) ‘hair loss’, (**B**) ‘dizziness’ and (**C**) ‘POTS’. Each final node shows a predicted class (0 or 1), the probability of that class, and what fraction of the dataset falls into that subgroup. Each terminal node shows the predicted class (0 = symptom absent, 1 = symptom present), the probability of that class, and the percentage of the dataset in that subgroup. For each split: “yes” means the condition is true, “no” means it is false. For example, “age < 52: yes” means age is less than 52 years; “no” means age is 52 or older. Gender is coded as 0 = female, 1 = male.

A decision tree could be created for the clinical symptoms, ‘hair loss’, ‘dizziness’, and ‘POTS’, yet not for the other clinical symptoms (Figure 3A–C).

These decision trees suggest, which biological markers (antibodies, age, gender) are most influential for each symptom in the dataset. The clinical symptom ‘hair loss’ is primarily separated by gender, then age. The clinical symptom ‘dizziness’ hinges almost entirely on age splits, whereas the clinical symptom ‘POTS’ uses a mix of GPCR-fAAb (β2-fAAb, M2-fAAb and α1-fAAb) and age.

**Figure 4:**
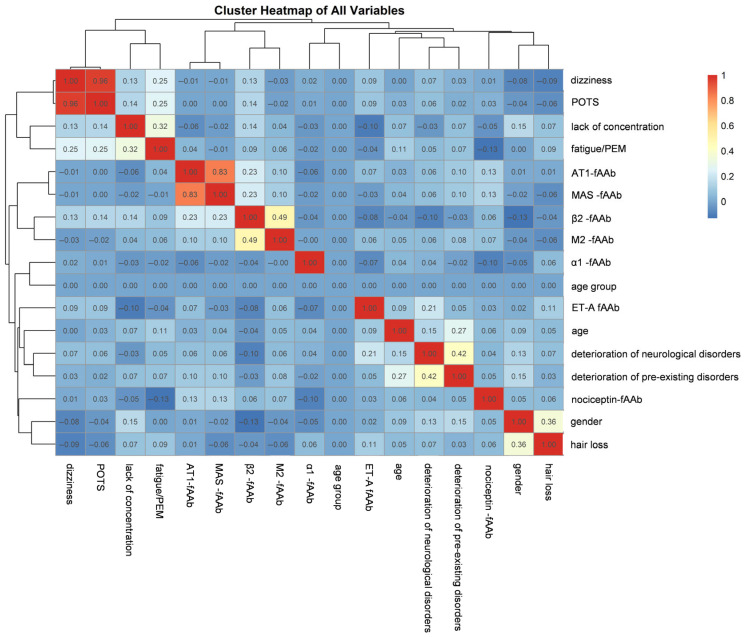
Cluster Heatmap of the present cohort including GPCR-fAAb and the self-reported symptoms, age and gender: Nociceptin-fAAb, β2-fAAb, AT1-fAAb, α1-fAAb, MAS-fAAb, M2-fAAb, ET-A-fAAb; self-reported symptoms: lack of concentration, dizziness, hair loss, Postural Orthostatic Tachycardia Syndrome (POTS), deterioration of pre-existing disorders, deterioration of neurological disorders, fatigue/post-exertional malaise (PEM). The variables appear along both the *x*- and *y*-axes, and each cell in the central matrix indicates the strength and direction of the association between the corresponding row variable (*y*-axis) and column variable (*x*-axis). The color scale on the right ranges from negative (blue) through neutral/low (white or light shades) to positive (red) association values. Darker shades of red denote stronger positive correlations, whereas darker shades of blue denote stronger negative correlations.

Additionally, the clustered heatmap (Figure 4) displays the pairwise correlations among all the studied variables, including gender and age. Hierarchical clustering dendrograms are placed along the top and left edges of the heatmap. These dendrograms group variables based on the similarity of their correlation profiles: those that tend to be positively or negatively correlated with the same sets of variables cluster more closely. Diagonal cells (variable vs. itself) are shown in bright red, representing perfect correlations of 1.0. By examining the color blocks and the branch lengths in each dendrogram, it is possible to visually identify variable groupings (‘lack of concentration’ and ‘fatigue/PEM’) that share similarities, as well as pairs or clusters of variables that exhibit notably high or low correlations. Among the GPCR-fAAb, β2-fAAb and M2-fAAb also form a tight cluster, indicating a strong positive correlation and possibly shared mechanistic roles or occurrence patterns.

Logistic regression output (including all GPCR-fAAb in the model) showed a significant relation between β2-fAAb and the clinical symptom ‘dizziness’ (*p*-value = 0.015, CI: [1.45, 31.59]). The positive estimate (1.91) indicates that individuals with a seropositivity of β2-fAAb are more likely to experience this symptom. After adjusting for confounding variables, the presence of the specific predictor variable (β2-fAAb) is significantly associated with a 6.77 times higher odds of experiencing the outcome (‘dizziness’) compared to its absence. In addition, a significant relation between the symptom β2-fAAb and the symptom ‘lack of concentration’ (*p*-value = 0.014, CI: [1.50, 37.07], estimate 2.0) and POTS (*p*-value = 0.0126, CI: [1.52, 32.17], estimate 1.94) were observed, respectively. After adjusting for confounding variables, the presence of the specific predictor variable (β2-fAAb) is significantly associated with 7.45 times higher odds of experiencing the outcome (‘lack of concentration’) and 6.99 for ‘POTS‘ compared to its absence. A seropositivity of ET-A-fAAb was significantly related to the symptom ‘deterioration of pre-existing neurological disorders’ (*p*-value = 0.044, CI: [0.08, 5.69]). The confidence interval suggests that this association is statistically significant, and the lower limit provides a lower bound for the strength of the association. Odds ratio was 16.67.

**Figure A1:**
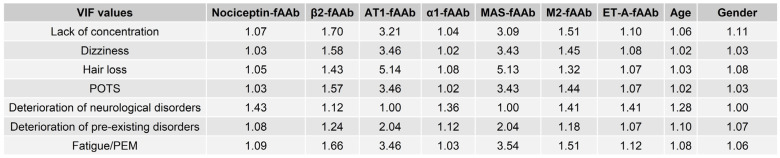
Variance Inflation Factor (VIF) values of GPCR-fAAb and self-reported clinical symptoms; most of the values are around 1, suggests little to no multicollinearity. Except for hair loss and AT1-fAAb and MAS-fAAb, there are no higher values (usually above 5 or 10) and this indicates the potential absence of problems with redundant information among the predictors that might affect the reliability of the regression estimates.

A subsequent analysis was conducted to evaluate the interaction between M2-fAAb and β2-fAAb, with fatigue/PEM designated as the outcome variable. The interaction term was not statistically significant, indicating no strong evidence that the effect of M2-fAAb on fatigue/PEM is modified by β2-fAAb levels. In a model restricted to the main effects of M2-fAAb and β2-fAAb, both β2-fAAb and gender were significantly associated with fatigue/PEM at the 5% significance level (*p* = 0.02 for each). Variance Inflation Factor (VIF) to assess multicollinearity for each predictor can be seen in Figure A1.

## 3. Discussion

LC has a multifaceted clinical phenotype, characterized by various clinical symptoms, thus diverse molecular pathways can be assumed. One of these LC pathways seems to be mediated by a specific autoimmune pattern [26]. Classical autoimmunity is based on a loss of self-tolerance, which is based on each individuals’ epigenetics and genetics, being modified by the external factors (e.g., life style, environment) [38,39]. The innate und adaptive immune response interact in these classical molecular mechanisms, generating autoantibodies and finally apoptosis [40]. In addition to this well-known autoimmune response, a different type of AAb was observed in Grave’s disease and dilatative cardiomyopathy [41,42,43]. These AAb did not induce apoptosis. They can activate their targets and thus change the receptor mediated physiologically cellular response in a pathologically way, therefore called fAAb. Contrary to physiological agents these fAAb-mediated response is characterized by a prolonged desensibilization of the receptors and lack of receptor downregulation [27,44]. This special type of AAb was observed in systemic and local disorders previously, most of them involving the cardiovascular system. As a recent study described a seropositivity of GPCR-fAAb in patients with PCS, it was the aim of the present study to investigate a potential clustering of GPCR-fAAb and their association to specific clinical symptoms in patients with LC. A total of 194 sera of patients with LC were tested by the established cardiomyocyte-bioassay for analysis of the presence and functionality of the GPCR-fAAb [45]. A pattern of β2-fAAb, M2-fAAb, AT2-fAAb and MAS-fAAb was observed to be the most common ones in the study cohort. Especially, β2-fAAb, was observed to be linked to the clinical symptom ‘dizziness’, ‘lack of concentration’ and POTS. As β2-fAAb and M2-fAAb were observed to form a tight cluster, a strong positive correlation was assumed. In the statistical model restricted to the main effects of β2-fAAb and M2-fAAb, both β2-fAAb and gender were significantly associated with fatigue/PEM. Interestingly, the β2-fAAb does not modify the M2-fAAb response on that clinical symptom.

The present study identified a cluster of β2-fAAb, M2-fAAb, AT1-fAAb and MAS-fAAb as most common feature in this study cohort. Clustering of different GPCR-AAbs seems to be present in several disorders: Patients with POTS showed seropositivities of β1-fAAb, β2-fAAb, α1-fAAb, M2-fAAb, and AT1-fAAb [44,46,47,48]; distinct patterns including β2-fAAb and M2-fAAb were observed in Chagas cardiomyopathy, electrical cardiac abnormalities, complex regional pain syndrome and fatigue syndrome [35,49,50,51]. A combination of α1-fAAb and AT1-fAAb was reported in Thromboangiitis obliterans [52]. A slight age-trend was observed for Noci-, MAS-, M2- and AT1- fAAb, potentially arguing for the well-known feature in classical autoimmunity that dysregulation can be followed by autoimmunity in older ages [44]. In addition, recent data demonstrate that age is associated with increased natural autoantibody production, being further amplified in patients with severe COVID-19 [53].

Considering gender as cofactor, the clinical symptoms, ‘hair loss’, ‘lack of concentration’ and ‘deterioration of pre-existing conditions’ were significantly more common symptoms in female patients than in male patients, potentially due to hormonal factors [54,55].

The way and timepoint for generation of GPCR-fAAb is still elusive. Classical AAbs targeting Angiotensin II (AngII), Angiotensin Converting Enzyme 2 (ACE2) and MAS1 oncogene (MAS) receptors were frequently observed in COVID-19 patients, suggesting that the production of GPCR-fAAb may begin even at the time of acute COVID-19 infection [56,57,58]. The virus enters cells through the RAS system and changes their affinity for their ligands [59]. In addition, the high intensity of the immune response to viral infections may also contribute to the production of GPCR-fAAb, potentially by molecular mimicry. However, as the virus is eliminated by the immune response, GPCR-fAAb persist, building up a cyclical process involving continuous GPCR-fAAb production, receptor overstimulation, increased intra- and extracellular oxidative stress and ultimately microcirculatory dysfunction, consequently resulting in LC symptoms [56].

The most common GPCR-fAAb, observed in patients with LC were β2-fAAb, M2-fAAb, MAS-fAAb and AT1-fAAb. It can be assumed that the molecular pathways of that four GPCR-fAAb might interact: β2-fAAb showed a link to an impaired capillary microcirculation in local and systemic disorders [27,36,37,60,61]. In addition, AT-1 receptor mediated signals can trigger vasoactive processes via, e.g., vasoconstriction [26]. It has been shown that AT1-fAAb can increase intracellular reactive oxygen species (ROS) production in human vascular smooth muscle cells, probably due to increased NADPH oxidase activity [62]. Increased ROS can induce DNA damage, triggering the activation of a DNA repair enzyme called poly-ADP-ribose polymerase-1 (PARP1) in endothelial cells [63]. In addition, research data showed that the AT1-fAAb mediated effect on renal arteries is intensified in ischemic or inflammatory tissues, whereas only a moderate effect was observed on renal arteries with good oxygen supply [37]. Of interest, M2-fAAb triggers the production of prostaglandin E2 and nitric oxide synthase, and induce the expression of cyclooxygenase 2 and inducible NOS mRNA, consequently inducing proinflammatory signals [64]. Further on, M2-fAAb was observed to inhibit the L-type Ca^2+^ current, increase cyclic GMP and the outward potassium current [65]. MAS-fAAb can reduce the effects of angiotensin II [66,67].

**Figure 5:**
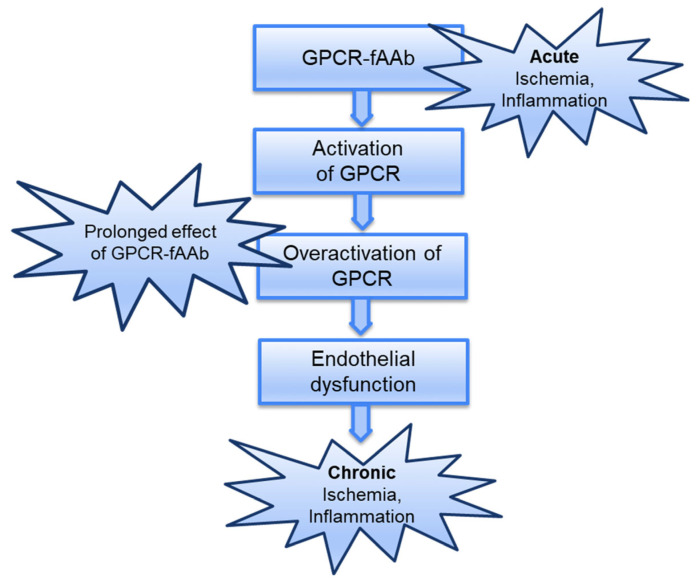
Proposed GPCR-fAAb mediated mechanism: we assume that GPCR-fAAb act as pathogenic agonistic AAbs by inducing a permanent (i.e., chronic) activation of their targets; these GPCR-fAAb-mediated pathogenetic effects were enhanced in ischemic/inflammatory tissues, inducing a pathogenetic circulus of inflammation and ischemia.

Summarizing this, it can be assumed that GPCR-fAAb act as pathogenic agonistic AAbs by (I) inducing a permanent activation of their targets, which (II) lose their mechanisms for protection against overstimulation; these GPCR-fAAb-mediated effects were (III) enhanced in ischemic/inflammatory tissues, inducing a (IV) pathogenetic circulus of inflammation and ischemia (Figure 5).

The present study is not without limitations. The study was designed as cross-sectional study; thus, it would be of interest if a seropositivity of GPCR-fAAb would change over time, potentially also associated with the clinical LC-symptoms. In addition, as study participants were adults, a clinical study, investigating the presence of GPCR-fAAb in patients with LC, being younger than 18 years, would be of interest.

## 4. Materials and Methods

### 4.1. Participants

A prospective study was performed at the Department of Ophthalmology, University of Erlangen, Friedrich-Alexander-Universität Erlangen-Nürnberg (FAU): 194 patients with LC (female: *n* = 106, age M = 41.35; SD = 12.95; male: *n* = 88, age M = 39; SD = 13.5) were recruited. LC was defined as ongoing and/or new clinical symptoms after acute SARS-CoV-2 infection. Mean time after acute COVID-19 was 325 days (SD = 173). The autoimmune LC’s subgroup with a seropositivity of GPCR-fAAb was included in the present study. The study has been approved by the local ethics committee and performed in accordance with the tenets of the Declaration of Helsinki. All patients signed a written informed consent form.

### 4.2. Measurements of GPCR-fAAb by the Cardiomyocyte (CM)-Bioassay

The cardiomyocyte bioassay established by Wallukat and Wollenberger [30,68] was used for the detection of GPCR-fAAb. Briefly, the hearts of newborn Wistar rats were removed and cells from the neonatal rat heart ventricles were isolated with a crude trypsin solution as described by Wallukat et al. [30]. After some digestion steps the cardiomyocytes were kept in culture. For the detection of GPCR-fAAb, the sera were dialyzed against a dialysis buffer of 0.15 M NaCl, 10 mM phosphate buffer at pH 7.4 (Membra-Cel MD 44, 14 kDa, SERVA, Heidelberg, Germany), to remove the pharmacological compounds, hormones and peptides. The sera were stored at −20° until further analysis. After determining the basal frequency of cardiomyocytes, IgG (1:50) was added to the cells and incubated for 60 min. Depending on the change in basal frequency (positive, negative chronotropic effect or no effect) with the use of specific receptor inhibitors, it was possible to specify which GPCR-fAAb the patient sera were positive for. The following inhibitors were used for the analysis 0.1 μM ICI118,551 (β2-fAAb), 1 µM atropin (M2-fAAb), 1 µM losartan (AT1-fAAb), 1 μM A779 (MAS-fAAb), 0.1 μM BQ123 (ETA-fAAb), 0.1 μM J113397 (Nociceptin-fAAb) and 1 μM urapidil or prazosin (α1-fAAb). After the addition of the specific inhibitor, the frequency was measured and the difference between the three measurements were analyzed. GPCR-fAAb were subdivided into a group, showing a positive chronotropic effect (β2-fAAb, α1-fAAB, AT1-fAAb; Nociceptin(-fAAb) or negative chronotropic effect (M2-fAAb, MAS-fAAb, ETA-fAAb). All materials, including solvents and chemical agents, were purchased from Merck (Darmstadt, Germany).

### 4.3. Statistics

Seropositivity of GPCR-fAAb was defined as independent variable in a series of logistic regression analyses. Different models were constructed, each corresponding to a specific symptom as the dependent variable. Age and gender were included as confounding variables to control their potential impact on the association between the GPCR-fAAb (predictors) and the presence of symptoms (outcome). This approach enabled the estimation of adjusted odds ratios (ORs), which represent the multiplicative change in the odds of experiencing a symptom associated with a one-unit increase in the level of a given GPCR-fAAb, holding age, gender, and the other antibodies constant. The analysis focused on identifying statistically significant associations by considering only those variables for which the *p*-values were significant (*p*-value was set significant if <0.05). This approach ensures that the reported associations are robust and less likely to be attributed to random chance. Due to the small sample size of the variable deterioration of neurological disorders, we used logistic regression with Firth’s penalized likelihood approach, to reduce small-sample bias. Additionally, the Variance Inflation Factor (VIF) values of GPCR-fAAb and self-reported clinical symptoms were calculated to check multicollinearity and the variables with VIF > 5 were excluded from final models. VIF_j_ = 1/1 − R^2^_j_ Rj^2^ is the coefficient of determination (R-squared) obtained when Xj is regressed on all the other predictor variables in the model.

The significance of the adjusted odds ratios provides valuable insights into the potential clinical relevance of the relationships between antibodies and specific symptoms, accounting for demographic factors. Together with the *p*-values we reported the Confidence Interval (CI). The adjusted odds ratio represents the change in the odds of the outcome (symptom) associated with a one-unit change in the predictor (GPCR-fAAb), holding other variables constant (the rest of GPCR-fAAb). The odds ratio represents the multiplicative change in the odds of the outcome associated with a one-unit increase in the predictor. An odds ratio greater than 1 suggests an increase in odds, while an odds ratio less than 1 suggests a decrease; a positive coefficient suggests a positive association, it implies that the presence of that GPCR-fAAb is associated with an increase in the log-odds of the outcome variable (symptom). Individuals with this GPCR-fAAb are more likely to experience the symptoms compared to those without the antibody. Contrary, negative coefficients suggest that individuals with this GPCR-fAAb are less likely to experience the symptoms compared to those without the GPCR-fAAb.

In addition to regression modeling, a hierarchical clustering dendrogram was performed to explore the correlation structure among variables. The dendrogram group together variables with similar overall correlation patterns: those that tend to be positively or negatively correlated with the same sets of variables cluster more closely.

To complement the analysis and decision tree analyses, analyses were performed for each symptom. The outcome variable was recoded as a factor with levels ‘0′ and ‘1.’ Predictors comprised the antibody variables, Age, and Gender. The dataset was split into training (70%) and test (30%) sets using the createDataPartition function from the caret package with a complexity parameter (cp) using cross-validation to identify the optimal value that balances model complexity and predictive performance, rather than fixing it at an arbitrary value. This approach reduces the risk of overfitting and underfitting. Model performance was evaluated on the independent test set. Predictions were generated in both class and probability formats. Classification accuracy was assessed using a confusion matrix, sensitivity and specificity. The final decision tree was visualized to interpret the hierarchical structure of predictive variables. All analyses and plots were produced in RStudio (Posit Software, Boston, MA, USA; version 2023.09.1 Build 494, ©2009–2023, PBC) using stats and base graphics functions. The following packages were applied: for data rearrangement and extraction: dplyr; ggplot2; carData and caret. The package for modeling and data evaluation were: rpart; randomForest; for data visualization: corrplot; ggplot2; pheamap; lattice and rpart.plot.

## 5. Conclusions

**Figure 6:**
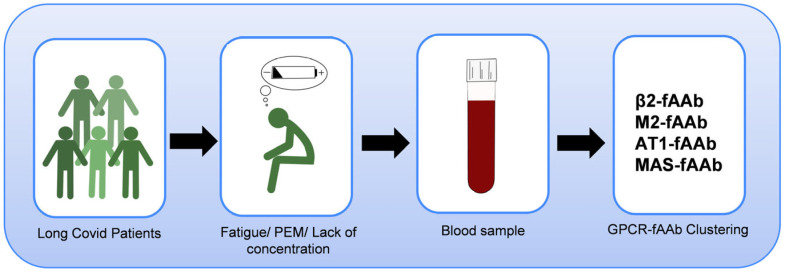
Major clinical phenotype with specific GPCR-fAAb cluster of the present study cohort: a cluster of β2-fAAb, M2-fAAb, MAS-fAAb and AT1-fAAb was the most common one in LC-patients with fatigue/PEM as one major clinical symptom.

Patients with LC were diagnosed with a wide range of symptoms. Serological analysis yielded that a cluster of β2-fAAb, M2-fAAb, MAS-fAAb and AT1-fAAb was the most common one in LC-patients with fatigue/PEM as one major clinical symptom (Figure 6). As additionally, a strong positive correlation between β2-fAAb and M2-fAAb and an association of β2-fAAb and gender to the main self-reported clinical symptom fatigue/PEM were observed, we assume a critical impact of GPCR-fAAb on LC-pathogenesis.

## Data Availability

The datasets generated and/or analyzed during the current study are available from the corresponding author on reasonable request.

## Abbreviations

LCS	Long-COVID Syndrome
PCS	Post-COVID Syndrome
SARS-CoV2	Severe acute respiratory syndrome coronavirus 2
COVID-19	Coronavirus Disease 2019
PEM	Post-exertional malaise
POTS	Postural orthostatic tachycardia syndrome
AAb	Autoantibodies
GPCR-fAAb	G-Protein coupled receptor autoantibodies with functional activity
β1-fAAb	Functional autoantibodies targeting the Beta-1 Adrenergic receptor
β2-fAAb	Functional autoantibodies targeting the Beta-2 Adrenergic receptor
CFS	Chronic fatigue syndrome
M2-fAAb	Functional autoantibodies targeting the Muscarinergic M2 receptor
AT1-fAAb	Functional autoantibodies targeting the Angiotensin II Type 1 receptor
MAS-fAAb	Functional autoantibodies targeting the angiotensin (1–7) Mas receptor
α1-fAAb	Functional autoantibodies targeting the Alpha-1 Adrenergic receptor
Nociceptin-fAAb	Functional autoantibodies targeting the Nociceptin receptor
ETA-fAAb	Functional autoantibodies targeting the Endothelin Type A receptor
CI	Confidence Interval
VIF	Variance Inflation Factor
CM	Cardiomyocyte
RAS	Renin-angiotensin system
ROS	Reactive oxygen species
PARP1	Poly-ADP-ribose polymerase-1
CRPS	Complex regional pain syndrome
AngII	Angiotensin II receptor
ACE2	Angiotensin Converting Enzyme 2 receptor
MAS	MAS1 oncogene
IgG	Immunoglobulin G antibodies
OR	Odds ratio
cp	Complexity parameter
SD	Standard Deviation
M	Mean Deviation
FAU	Friedrich-Alexander-Universität Erlangen-Nürnberg
0	female
1	male
AA4	Alpha-1 Adrenergic Receptor functional autoantibodies
AA6	Muscarinergic M2 Receptor functional autoantibodes
NA	Missing value
